# Age-based scoring as a complementary approach to sustainable trophy hunting

**DOI:** 10.1093/biosci/biae091

**Published:** 2024-10-10

**Authors:** Jerrold L Belant, Kai-Uwe Denker, Kenneth F Kellner

**Affiliations:** Wild Foods Institute, Department of Fisheries and Wildlife at Michigan State University, East Lansing, Michigan, United States; African Hunting Safaris, Erongo Verzeichnis for African Game Animals, Namibian Professional Hunting Association, Windhoek, Namibia; Erongo Mountain Nature Sanctuary, Omaruru, Namibia; Wild Foods Institute, Department of Fisheries and Wildlife at Michigan State University, East Lansing, Michigan, United States

Hunting wildlife, particularly large mammals, is controversial, with divergent arguments that often conflate science and values. Hunting is done for many purposes, which are frequently considered synonymous even though variation in these purposes (e.g., recreation, subsistence) can be quite profound. The most controversial form of hunting is likely trophy hunting (Hare et al. 2023), whereby hunters legally harvest wildlife with a primary purpose being to retain one or more inedible parts of the killed animals (e.g., horns, tusks, skins; IUCN 2016). Although public support of hunting varies markedly on the basis of the purpose and which species is hunted, recent legislation has led to an import ban on hunting trophies from endangered species in 2024 to Belgium (Chini 2024) and a bill introduced in Great Britain that would prohibit the import of hunting trophies broadly aligned with species in appendices I and II of the Convention on International Trade in Endangered Species of Wild Flora and Fauna (Ares and Kelly 2023).

Hunting-based conservation organizations that measure harvested large mammals typically emphasize the maximum size of secondary sexual traits (e.g., horns, antlers, skull size), using a formal measurement and scoring system. For example, Safari Club International and the Rowland Ward Foundation provide widely accepted standards for measuring secondary sexual traits, and hunters that kill exceptionally large individuals receive higher scores and rankings (Safari Club International 2019, Rowland Ward Foundation 2024), often as a status symbol for those hunters. These systems consequently emphasize mature, breeding-age males, because horn or antler attrition in older males can reduce their overall size and desirability for harvest (Josling et al. 2019), although immature individuals can potentially achieve horn growth that exceeds minimum scoring standards for trophy status before participating in breeding.

Studies of North American ungulates have suggested associations between the long-term selective harvest of males and reduced horn size (Pérez et al. 2011) or age structure (Monteith et al. 2013, Schindler et al. 2016), but overall data are limited, more so for African ungulates. Horn and antler size are heritable and associated with fitness-related traits in males and females (Coltman et al. 2005) and can confer increased reproductive success (Monteith et al. 2018). The management strategies typically used to address potential adverse effects on populations from selective harvest include quotas limiting the number of animals that can be harvested on the basis of certain horn and antler sizes or the number of antler points. Age-based hunting, whereby males to be harvested must be of an approximate minimum age, has also recently been implemented. For example, in several African nations, only older male lions (*Panthera leo*), whose ages are estimated using external morphology (Miller et al. 2016), can be legally harvested, with the intent to harvest males no longer contributing to the viability and genetic composition of the lion populations.

Namibia has recently adopted a comprehensive age-related trophy measurement and scoring system for Bovidae, Elephantidae, Suidae, Hippopotamidae, Felidae, and Hyaenidae to provide greater recognition of harvested animals considered past their reproductive prime age. Developed by the Erongo Verzeichnis (registry) for African game animals, the goals of this system are to discourage the hunting of immature animals, to encourage the harvest of animals considered past their prime breeding age, to improve the role of hunting in conservation, to expand awareness of how trophies can be valued by considering animal age, and to aid management planning toward nondetriment or enhancement findings for importing authorities (Denker 2020). This system categorizes individuals into breeding age classes as immature, prime, or past prime whereby immature individuals are disqualified from scoring, measurement scores of prime individuals are multiplied by a factor of 1.0, and past prime individual measurements are multiplied by 1.12 to establish final scores (figure 1). In this system, a handicap score is used for those individuals past their prime breeding age such that their total score could meet or exceed that of prime or mature males. This scoring system is broadly aligned with the Rowland Ward system (e.g., using the longest horn) but incorporates additional measurements in species that exhibit pronounced tendencies for horn wear (e.g., brooming) or other age-related attributes (e.g., ornamental ring sections in *Alcelaphus, Hippotraqus*, and *Taurotragus* spp.; Denker 2020).

**Figure 1.**
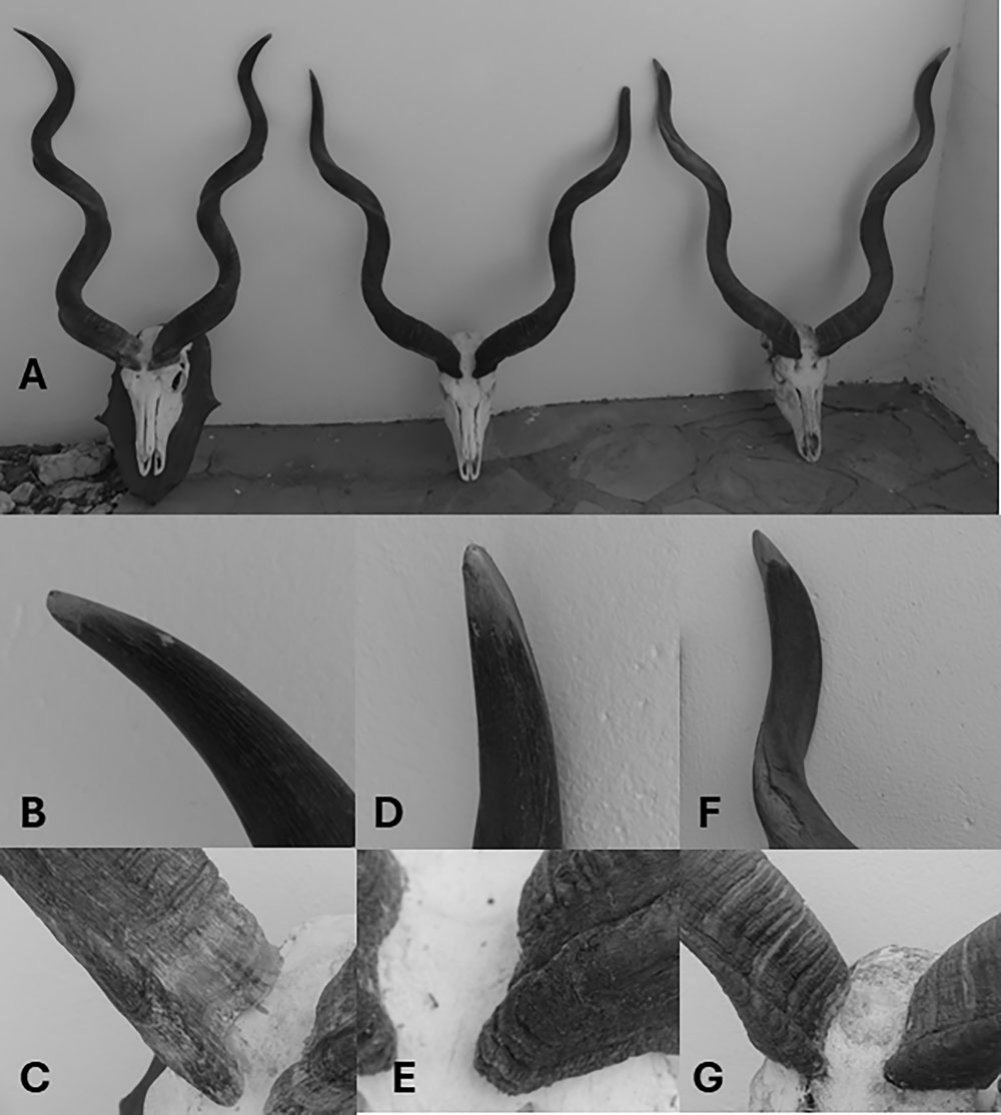
(A) Horns (left to right) of immature, prime, and past prime greater kudu (*Tragelaphus strepsiceros*); the immature individual would receive the greatest trophy measurement score. (B) Immature greater kudu horn tip showing limited wear and (C) soft, fibrous, lighter colored cells at the base of the horns. (D) Prime greater kudu horn tip showing increased wear and (E) darker, fully hardened and developed cells at the base of the horns. (F) Past prime greater kudu with more blunted horn tip, coarse flaking of dead horn cells, and (G) darker, hardened cells with inward growth at the base of the horns.

Since 2020, the Namibian Professional Hunting Association (NAPHA) has used the age-related trophy system to emphasize the harvest of past prime individuals and to recognize hunters for hunting older animals less likely to contribute to breeding populations (table 1). Basic NAPHA scoring thresholds have been developed for each species at bronze, silver, and gold levels. In addition, NAPHA has developed a top award (i.e., the Game Fields Medal) for trophies of past prime native species from freely breeding populations that measure 5% above the gold medal threshold. Furthermore, a Conservation Medal is awarded to individuals that harvest past prime individuals that do not meet other award criteria.

**Table 1.** Comparison of measurement systems used to score secondary traits (e.g., horns) of trophy-hunted animals.

Measurement system	Measurement categories	Handicap score	Potential for improved species conservation
Safari Club International	Single category	1.0	Reduced
Rowland Ward	Single category	1.0	Reduced
Age-related trophy	Immature age class	Discouraged	Increased
	Prime-age class	1.0	Reduced
	Past prime age class	1.12	Increased

*Note:* The handicap score represents multiplying factors used to adjust scores of final measurements. The potential outcomes for improved species (or population) conservation correspond with conceptual relative potential to maintain genetic diversity and population goals, particularly for populations where hunting demand is high.

Public perceptions of trophy hunting are improved when the economic benefits favor local communities and when conservation is improved (Hare et al. 2024). Namibia's age-related trophy system could further improve public perception and support of hunting, because the intent is to increase hunter acceptance and harvest of individuals considered no longer contributing to species recruitment. This, in turn, could improve societal acceptance of trophy hunting, particularly when coupled with the donation of meat to local communities (e.g., White and Belant 2015).

We do not suggest that existing measurement and scoring systems are inherently wrong, because each is based on specific goals and objectives. We also acknowledge that there are multiple harvest management strategies available to achieve intended harvest and conservation objectives. However, we suggest that the age-related trophy model has potential to complement existing systems and further benefit the conservation of harvested species and maintain the corresponding ecosystem services (e.g., hunting, food consumption) and processes (e.g., genetic diversity, predator–prey relations), as well as enhancing public perceptions of hunting. Furthermore, the age-related trophy system could have greater application with species or populations that are limited in numbers relative to hunting interest as past prime age individuals could be emphasized on the basis of desirability for harvest or incorporated into an overall larger total harvest quota. Namibia is presently planning an initial evaluation of the age-related trophy system, but this evaluation will require rigorous and quantitative assessments among age classes and scoring measurements across species. Although species attributes (e.g., horn length) can vary with age in some African ungulates (Crosmary et al. 2013), age-based data are limited. Furthermore, research on the contributions of various age classes to reproduction and genetic diversity, along with the effects of reducing certain age classes (e.g., prime-age individuals) on these factors is urgently needed. Whether this more comprehensive approach to age-based harvest management will achieve its desired goals will require long-term monitoring of age-related trophy measurements along with wildlife population age structure, abundance, and genetic diversity in a semiexperimental approach using free-ranging populations across diverse species.

## Data Availability

No data was used in the development of this manuscript.

## Author Biography

Jerrold L. Belant (jbelant@msu.edu) and Kenneth F. Kellner are affiliated with the Wild Foods Institute, in the Department of Fisheries and Wildlife at Michigan State University, in East Lansing, Michigan, in the United States. Kai-Uwe Denker is affiliated with African Hunting Safaris, Erongo Verzeichnis for African Game Animals, at the Namibian Professional Hunting Association, in Windhoek, Namibia and with Erongo Mountain Nature Sanctuary, in Omaruru, Namibia.

## References

[bib1] Ares E , Kelly R. 2023. Hunting Trophies (Import Prohibition) Bill 2023–2024. House of Commons Library, UK Parliament. https://commonslibrary.parliament.uk/research-briefings/cbp-9991.

[bib2] Chini M . 2024. Belgium bans import of hunting trophies from endangered species. Brussels Times (26 January 2024). www.brusselstimes.com/894881/belgium-bans-import-of-hunting-trophies-from-endangered-species.

[bib3] Coltman DW , O'Donoghue P, Hogg JT, Festa-Bianchet M. 2005. Selection and genetic (co)variance in bighorn sheep. Evolution: International Journal of Organic Evolution 59: 1372–1382. 16050112

[bib4] Crosmary WG , Loveridge AJ, Ndaimani H, Lebel S, Booth V, Cóte SD, Fritz H. 2013. Trophy hunting in Africa: Long-term trends in antelope horn size. Animal Conservation 16: 648–660.

[bib5] Denker K ed. 2020. Erongo Verzeichnis für Afrikanisches Jagdwild. Omaruru.

[bib6] Hare D et al. 2023. Trophy hunting is not one big thing. Biodiversity and Conservation 32: 2149–2153.

[bib7] Hare D , Dickman AJ, Johnson PJ, Rono BJ, Mutinhima Y, Sutherland C, Kulunge S, Sibanda L, Mandoloma L, Kimaili D. 2024. Public perceptions of trophy hunting are pragmatic, not dogmatic. Proceedings of the Royal Society B 291: 20231638. 38351797 10.1098/rspb.2023.1638 PMC10865007

[bib8] [IUCN] International Union for Conservation of Nature . 2016. Informing decisions on trophy hunting. IUCN. https://wwfint.awsassets.panda.org/downloads/iucn_informingdecisionsontrophyhuntingv1_1.pdf.

[bib9] Josling GC , Lepori AA, Neser FWX, Lubout PC, van Wyk JB. 2019. Evaluating horn traits or economic importance in sable antelope (*Hippotragus niger niger*). South African Journal of Animal Science 49: 40–49.

[bib10] Miller JRB et al. 2016. Aging traits and sustainable trophy hunting of African lions. Biological Conservation 201: 160–168.

[bib11] Monteith KL , Long RA, Belich VC, Heffelfinger JR, Krausman PR, Bowyer RT. 2013. Effects of harvest, culture, and climate on trends in size of horn-like structures in trophy ungulates. Wildlife Monograph 183: 1–26.

[bib12] Monteith KL , Long RA, Stephenson TR, Bleich VC, Bowyer RT, Lasharr TN. 2018. Horn size and nutrition in mountain sheep: Can ewe handle the truth? Journal of Wildlife Management 82: 67–84.

[bib13] Pérez JM et al. 2011. Reduced horn size in two wild trophy-hunted species of Caprinae. Wildlife Biology 17: 102–112.

[bib14] Rowland Ward Foundation . 2024. Rowland Ward's Measuring Handbook. Rowland Ward.

[bib15] Safari Club International . 2019. Official Measurer's Manual. Safari Club International.

[bib16] Schindler S , Festa-Bianchet M, Hogg JT, Pelletier F. 2016. Hunting, age structure, and horn size distribution in bighorn sheep. Journal of Wildlife Management 81: 792–799.

[bib17] White PA , Belant JL. 2015. Provisioning of game meat to rural communities as a benefit of safari hunting in Zambia. PLOS ONE 10: e0117237. 25693191 10.1371/journal.pone.0117237 PMC4334497

